# Association between remnant cholesterol and chronic kidney disease in Chinese hypertensive patients

**DOI:** 10.3389/fendo.2023.1189574

**Published:** 2023-06-21

**Authors:** Ting Yuan, Congcong Ding, Yanyou Xie, Xinlei Zhou, Chong Xie, Tao Wang, Chao Yu, Wei Zhou, Lingjuan Zhu, Huihui Bao, Xiaoshu Cheng

**Affiliations:** ^1^ Department of Cardiology, The Second Affiliated Hospital of Nanchang University, Nanchang, Jiangxi, China; ^2^ Center for Prevention and Treatment of Cardiovascular Diseases, The Second Affiliated Hospital of Nanchang University, Nanchang, Jiangxi, China; ^3^ Jiangxi Provincial Cardiovascular Disease Clinical Medical Research Center, Nanchang, Jiangxi, China; ^4^ Jiangxi Sub-center of National Clinical Research Center for Cardiovascular Diseases, Nanchang, Jiangxi, China

**Keywords:** remnant cholesterol, chronic kidney disease, Chinese hypertensive population, cross-sectional study, lipid metabolism

## Abstract

**Background:**

Remnant cholesterol (RC) and chronic kidney disease (CKD) have not been definitively linked in individuals with different characteristics. This study aims to investigate the relationship between serum RC level and CKD and examine possible effect modifiers in Chinese patients with hypertension.

**Methods:**

Our study is based on the Chinese H-type Hypertension Project, which is an observational registry study conducted in real-world settings. The outcome was CKD, defined as an estimated glomerular filtration rate of less than 60 ml/min·1.73 m^2^. Multivariate logistic regression and smooth curve fitting were used to analyze the association between RC and CKD. Subgroup analyses were subsequently conducted to examine the effects of other variables.

**Results:**

The mean age of the 13,024 patients with hypertension at baseline was 63.8 ± 9.4 years, and 46.8% were male. A conspicuous linear positive association was observed between RC level and CKD (per SD increment; odds ratio [OR], 1.15; 95% confidence interval [CI], 1.08–1.23). Compared with the lowest quartile group of RC, the risk of CKD was 53% higher (OR, 1.53; 95% CI, 1.26–1.86) in the highest quartile group. Furthermore, a stronger positive association between RC level and CKD was found among participants with a higher body mass index (BMI <24 *vs*. ≥24 kg/m^2^; *P*-interaction = 0.034) or current non-smokers (smoker *vs*. non-smoker; *P*-interaction = 0.024).

**Conclusions:**

Among Chinese adults with hypertension, RC level was positively associated with CKD, particularly in those with a BMI of ≥24 kg/m^2^ and current non-smokers. These findings may help improve lipid management regimens in patients with hypertension.

## 1 Introduction

Chronic kidney disease (CKD) has high morbidity and mortality, particularly among people with diabetes and hypertension (1). By 2040, CKD is predicted to become the fifth leading cause of mortality worldwide (2). Common factors promoting the occurrence and development of CKD include diabetes, hypertension, dyslipidemia, and smoking (3). Lipid metabolism disorders are common in patients with CKD. Previous studies have shown various lipid concentrations and structural changes, including higher triglyceride (TG) levels, in patients with CKD (4, 5). Nevertheless, most of the existing research is focused on traditional lipid profiles.

Remnant cholesterol (RC) is a recently described lipid indicator. It is the cholesterol component of triglyceride-rich lipoproteins, consisting of cholesterol cargo of very low-density lipoprotein (VLDL) and medium-density lipoprotein (IDL) in the fasting state and chylomicron (CM) remnants in the non-fasting state (6). Previous research has shown that recurrent atherosclerotic cardiovascular disease (ASCVD) can occur even when the low-density lipoprotein cholesterol (LDL-C) concentration is reduced to an optimal level. This residual risk is believed to be caused by remnant cholesterol (RC) (7, 8). As a new cholesterol index, in the past ten years, RC has been confirmed by extensive research to be related to the initiation and progression of atherosclerosis and ASCVD (9–11).

Previous studies have demonstrated inconsistent results regarding the association between RC levels and CKD. Some studies have shown that elevated levels of RC or its components (comprising VLDL and IDL cholesterol) are associated with an increased risk of CKD, whereas others have not found this association. However, the hypertensive population is unique and is a high-risk subgroup for CKD. A discussion of the relationship between RC and CKD in patients with hypertension may provide a new direction for lipid management in populations with hypertension in the future. Therefore, the association between RC levels and CKD risk in a Chinese population with hypertension needs to be assessed.

## 2 Materials and methods

### 2.1 Study population and design

This cross-sectional study used observational data from the Chinese hypertension Registry. The study was conducted in July 2018 in Wuyuan, Jiangxi Province, China. A total of 14,268 participants aged ≥18 years were included after excluding those who were unable to provide informed consent or failed to complete follow-up for various reasons. Patients with hypertension are defined as those with systolic blood pressure (SBP) ≥140 mmHg and/or diastolic blood pressure (DBP) ≥90 mmHg with a previous diagnosis of hypertension or currently taking antihypertensive medication. This study was approved by the Ethics Committee of the Biomedical Institute of Anhui Medical University, and written informed consent was obtained from all study participants.

Among the 14,268 participants, we excluded those without hypertension (n = 34), those with missing data for total cholesterol (TC), high-density lipoprotein cholesterol (HDL-C), LDL-C (n = 7), use of lipid-lowering drugs (n = 506), and individuals with extreme values of RC (n = 697). Ultimately, 13,024 participants were included in the final analysis (**Figure S1**).

### 2.2 Data collection

We collected basic information on all the participants, including sex, age, height, weight, waist circumference (WC), drinking status, and smoking status. All blood samples were collected by professionals to examine fasting TC, TG, HDL-C, LDL-C, aspartate aminotransferase (AST), alanine transaminase (ALT), plasma homocysteine (Hcy), fasting blood glucose (FBG), and serum creatinine levels. All parameters were tested using a professional instrument (Beckman Coulter) at the Biaojia Biotechnology Laboratory, Shenzhen, China.

Based on the previous description, the RC was calculated as RC = TC - (HDL-C) - (LDL-C). CKD was defined as an estimated glomerular filtration rate (eGFR) of less than 60 ml/min·1.73 m^2^. The eGFR was calculated according to the Chronic Kidney Disease Epidemiology Collaboration (12). Body mass index (BMI) was calculated by dividing the weight by the square of height.

### 2.3 Covariates

The selected covariates related to CKD included sex, age, WC, BMI, DBP, drinking status, smoking status, SBP, Hcy, TG, diabetes, coronary heart disease (CAD), stroke, and use of antihypertensive drugs.

### 2.4 Statistical analysis

For continuous variables, we used means with standard deviations (SDs) or median (interquartile range), and categorical variables included the characteristics of the study population (percentages). To demonstrate the relationship between RC levels and CKD more intuitively, we used a generalized additive model and smooth curve fitting (penalty-spline method). Multivariate logistic regression was used to evaluate the odds ratios (ORs) and 95% confidence intervals (CIs) for CKD in RC (as a continuous variable and quartiles). We established two models in which the fully adjusted model (Model II) was adjusted for sex, age, WC, BMI, drinking status, smoking status, SBP, DBP, diabetes, CAD, stroke, antihypertensive drugs, Hcy, and TG. Subgroup and interaction analyses were used to further explore the potential effect modifiers. A sensitivity analysis was performed to confirm the robustness of the relationship between RC levels and CKD. All statistical analyses were performed using the statistical package R (http://www.R-project.org) and EmpowerStats (http://www.empowerstats.com, X&Y Solutions, Inc., Boston, MA), and a two-tailed *P <*0.05 was considered statistically significant.

## 3 Results

### 3.1 Baseline characteristics of the study population

The baseline characteristics of the 13,024 participants are described in **Table 1**. Overall, their average age was 63.8 ± 9.4 years, and 6,099 participants were male, accounting for 46.8%. The baseline characteristics of the study participants were stratified according to RC quartiles. The RC ranges for the quartiles were ≤0.41 mmol/L, 0.41–0.63 mmol/L, 0.63–0.83 mmol/L, and ≥0.83 mmol/L. Compared with the lowest quartile of RC, the population in the highest quartile mostly comprised older female participants who did not smoke or drink at the time and were more likely to have a history of diabetes mellitus. The values of BMI, WC, TC, SBP, LDL-C, FBG, TG, and UA were also higher. HDL-C level and eGFR were lower, and a smaller proportion of people took antihypertensive drugs.

**Table 1:** Characteristics of study participants by RC.

Variables	Total	RC, mmol/L				*P* value
		Q1 (<0.41)	Q2 (0.41-0.63)	Q3 (0.63-0.83)	Q4 (≥0.83)	
N	13024	3150	3319	3205	3350	
Age, years	63.8 ± 9.4	63.9 ± 9.1	63.7 ± 9.6	64.1 ± 9.3	63.4 ± 9.6	0.037
Male, n (%)	6099 (46.8)	1628 (51.7)	1722 (51.9)	1436 (44.8)	1313 (39.2)	<0.001
BMI, kg/m2	23.6 ± 3.7	23.3 ± 4.4	23.4 ± 3.5	23.7 ± 3.5	24.0 ± 3.5	<0.001
WC, cm	83.8 ± 9.8	82.9 ± 10.0	83.4 ± 9.7	84.1 ± 9.8	84.8 ± 9.8	<0.001
SBP, mmHg	148.5 ± 17.8	149.3 ± 17.7	147.7 ± 17.8	147.8 ± 17.6	149.4 ± 18.1	<0.001
DBP, mmHg	89.1 ± 10.8	88.8 ± 10.6	88.9 ± 10.8	89.0 ± 10.7	89.5 ± 11.0	0.095
Education status, n (%)						0.014
Primary school graduate or below	8571 (80.0)	1509 (80.7)	2216 (78.6)	2376 (80.5)	2470 (80.3)	
Middle/high/special school	2088 (19.5)	356 (19.0)	579 (20.5)	568 (19.2)	585 (19.0)	
College graduate or above	58 (0.5)	6 (0.3)	24 (0.9)	7 (0.2)	21 (0.7)	
Current smoking, n (%)	3335 (25.6)	865 (27.5)	905 (27.3)	790 (24.7)	775 (23.1)	<0.001
Current alcohol drinking, n (%)	2817 (21.6)	757 (24.0)	730 (22.0)	649 (20.3)	681 (20.3)	<0.001
Laboratory results						
Total cholesterol, mmol/L	5.2 ± 1.1	4.7 ± 1.0	4.9 ± 0.9	5.3 ± 0.9	6.0 ± 1.1	<0.001
Triglycerides, mmol/L	1.8 ± 1.3	1.3 ± 0.6	1.5 ± 0.8	1.8 ± 1.0	2.6 ± 1.8	<0.001
HDL-C, mmol/L	1.6 ± 0.4	1.7 ± 0.4	1.6 ± 0.4	1.5 ± 0.4	1.5 ± 0.4	<0.001
LDL-C, mmol/L	3.0 ± 0.8	2.8 ± 0.8	2.8 ± 0.7	3.1 ± 0.7	3.3 ± 0.9	<0.001
Fasting glucose, mmol/L	6.2 ± 1.6	6.0 ± 1.3	6.0 ± 1.3	6.2 ± 1.6	6.6 ± 2.0	<0.001
Total homocysteine, μmol/L	15.0 (12.4-19.1)	15.2 (12.6-19.4)	14.8 (12.4-19.0)	14.8 (12.4-19.0)	15.0 (12.5-18.8)	0.054
Uric acid, μmol/L	420.0 ± 121.0	397.4 ± 113.4	415.6 ± 118.3	421.9 ± 122.8	443.9 ± 124.6	<0.001
eGFR, ml/min/1.73 m²	88.3 ± 20.1	91.4 ± 20.6	88.5 ± 19.8	87.4 ± 19.3	86.0 ± 20.2	<0.001
History of disease						
Diabetes mellitus, n (%)	2341 (18.0)	453 (14.4)	474 (14.3)	570 (17.8)	844 (25.2)	<0.001
Coronary heart disease, n (%)	609 (4.7)	129 (4.1)	147 (4.4)	150 (4.7)	183 (5.5)	0.058
Stroke, n (%)	788 (6.1)	197 (6.3)	211 (6.4)	180 (5.6)	200 (6.0)	0.598
Medication use, n (%)						
Antihypertensive drugs	8298 (63.7)	2107 (66.9)	2101 (63.3)	1980 (61.8)	2110 (63.0)	<0.001
Glucose-lowering drugs	635 (4.9)	147 (4.7)	137 (4.1)	155 (4.8)	196 (5.9)	0.011
Antiplatelet drugs	289 (2.2)	72 (2.3)	76 (2.3)	71 (2.2)	70 (2.1)	0.94

Data are expressed as mean ± SD or median (interquartile range) and numbers (percentage) as appropriate.

RC, remnant cholesterol; BMI, body mass index; WC, waist circumference; SBP, systolic blood pressure; DBP, diastolic blood pressure; eGFR, estimated glomerular filtration rate; HDL-C, high-density lipoprotein cholesterol; LDL-C, low-density lipoprotein cholesterol.

### 3.2 Association between RC and CKD

**Figure 1:**
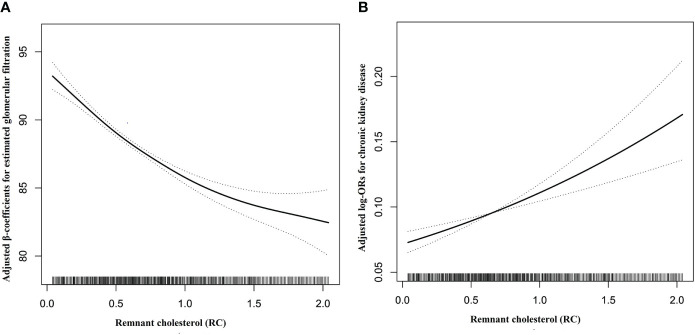
Dose-response relationships between RC and the risk of eGFR decline **(A)** and CKD **(B)**. Adjustment factors included age, sex, BMI, WC, SBP, DBP, TG, smoking status, drinking status, Hcy, diabetes, stroke, coronary heart disease, and antihypertensive drugs at baseline.

As shown in **Table 2**, multiple logistic regression models were constructed to assess the association between RC levels and CKD, and a positive association was observed. In a complete adjustment model, which adjusted for sex, age, WC, SBP, BMI, DBP, smoking status, drinking status; Hcy, TG, diabetes, stroke, CAD, and antihypertensive drugs, for each SD increment in RC, the risk of CKD increased by 15% (adjusted OR, 1.15; 95% CI: 1.08–1.23). Consistently, when serum RC level was assessed as quartiles, compared with the Q1, the adjusted ORs in the Q2, Q3, and Q4 were 1.12 (95% CI: 0.95–1.53), 1.15 (95% CI: 0.95–1.39), and 1.53 (95% CI: 1.26–1.86), respectively. Our research also found that compared with RC level <0.83mmol/L, RC level ≥0.83 mmol/L significantly increased the risk of CKD, (OR, 1.40; 95% CI: 1.21–1.62). In addition, we used a generalized additive model and smooth curve fitting (penalized spline method) to represent the association between RC levels and CKD. As shown in **Figure 1**, a linear relationship (*P* = 0.007) was observed between RC levels and CKD. We also analyzed the association between RC levels and eGFR. Moreover, the RC level was negatively associated with the eGFR. Further multiple linear regression analysis showed that for every SD increment in RC, eGFR decreased by 1.97 ml/min·1.73 m^2^ (95% CI: -2.29 to -1.65). Compared with the lowest quartile of RC, the eGFR of participants in the highest quartile decreased significantly (β = -6.35, 95% CI: -7.20 to -5.51) (**Table S1**).

**Table 2:** Association between RC and CKD in different models.

RC, mmol/L	Participants, n	Events, n (%)	Crude model		Model I		Model II	
			OR (95% CI)	*P* value	OR (95% CI)	*P* value	OR (95% CI)	*P* value
RC Z score	13024	1243 (9.5)	1.14 (1.09, 1.21)	<0.001	1.20 (1.13, 1.27)	<0.001	1.15 (1.08, 1.23)	<0.001
Quartiles								
Q1 (<0.41)	3150	257 (8.2)	Ref.		Ref.		Ref.	
Q2 (0.41-0.63)	3319	299 (9.0)	1.11 (0.94, 1.33)	0.223	1.12 (0.93, 1.34)	0.212	1.12 (0.93, 1.35)	0.245
Q3 (0.63-0.83)	3205	297 (9.3)	1.15 (0.97, 1.37)	0.118	1.17 (0.97, 1.40)	0.092	1.15 (0.95, 1.39)	0.156
Q4 (≥0.83)	3350	390 (11.6)	1.48 (1.26, 1.75)	<0.001	1.68 (1.41, 2.00)	<0.001	1.53 (1.26, 1.86)	<0.001
*P* for trend				<0.001		<0.001		<0.001
Categories								
Q1-Q3 (<0.83)	9674	853 (8.8)	Ref.		Ref.		Ref.	
Q4 (≥0.83)	3350	390 (11.6)	1.36 (1.20, 1.55)	<0.001	1.53 (1.34, 1.75)	<0.001	1.40 (1.21, 1.62)	<0.001

Crude model was adjusted for None; Model I was adjusted for age, sex, BMI, WC; Model II was adjusted for age, sex, BMI, WC, SBP, DBP, TG, smoking status, alcohol drinking status, Hcy, diabetes, stroke, coronary heart disease, antihypertensive drugs.

RC, remnant cholesterol; OR, odd ratio; 95% CI, 95% confidence interval, TG, triglycerides; Hcy, homocysteine.

### 3.3 Subgroup analyses

**Figure 2:**
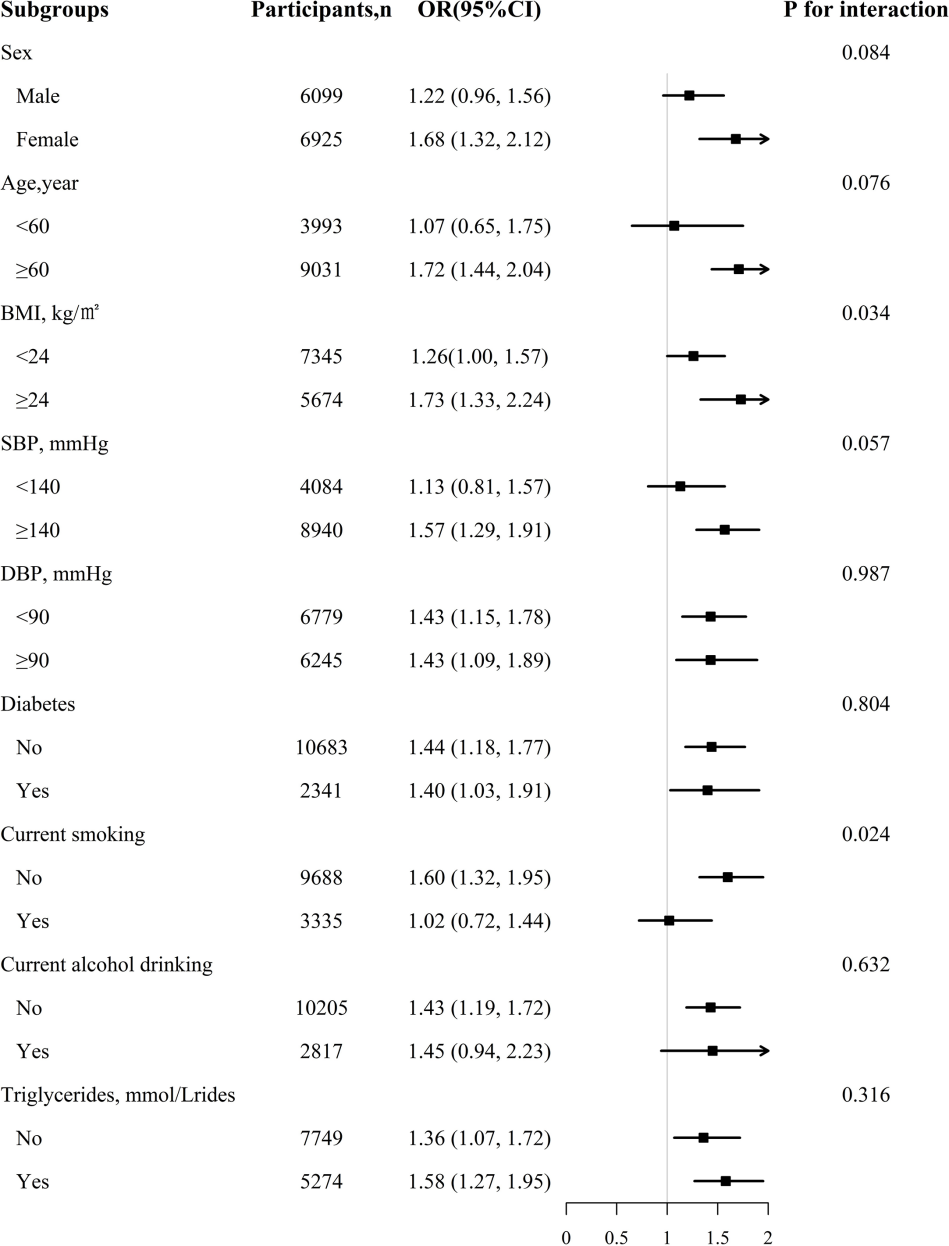
The association between RC and CKD in various subgroups. Each subgroup analysis adjusted, if not stratified, for age, sex, BMI, WC, SBP, DBP, TG, smoking status, drinking status, Hcy, diabetes, stroke, coronary heart disease, antihypertensive drugs at baseline.

To further explore the possible effect of modifiers on the relationship between RC levels and CKD, we performed a stratified analysis. In **Figure 2**, the association between RC level and increased CKD risk is significantly stronger in the subgroup of patients with BMI ≥24 kg/m^2^ than in those with BMI <24 kg/m^2^ and in non-smoking participants than in smoking patients (interaction *P*=0.034, 0.024 respectively). In participants with BMI ≥24 kg/m², for every unit increment in RC, the risk of CKD increased by 73% (OR, 1.73; 95% CI: 1.33–2.24; *P* = 0.034). Similarly, in current non-smokers, every additional unit of RC increased the risk of CKD by 60% (OR, 1.60; 95% CI: 1.32 1.95). However, sex, age, SBP, DBP, alcohol consumption status, diabetes, and antihypertensive drug use did not significantly modify the relationship between RC levels and CKD.

### 3.4 Sensitivity analysis

Owing to the close association between the RC and TG levels, the concentration of RC increased with an increase in the TG level (9, 13). Therefore, we carried out the sensitivity analysis, which explored the relationship between RC levels and CKD in populations with hypertension and normal TG levels. In **Table S2**, for each SD increment in RC, the risk of CKD increases by 11% (OR, 1.11; 95% CI: 1.02–1.19). Compared with Q1, the adjusted ORs in Q2, Q3, and Q4 were 1.17 (95% CI: 0.92–1.49), 1.11 (95% CI: 0.87–1.41), and 1.42 (95% CI: 1.12–1.79), respectively. In the previous analysis, given that RC was calculated from TC, LDL-C, and HDL-C, no adjustments were made in the model, but their collinearity with RC was considered to be large. In order to verify whether the relationship between RC and CKD obtained in this study is independent of traditional lipid indexes, residual analysis was conducted (**Table S3**). The positive association between RC and CKD risk still exists after adjusting for TC residuals, LDL-C residuals, and HDL-C residuals. This shows that the relationship between RC and CKD is independent of traditional lipid indexes.

## 4 Discussion

In this study, we found that the RC level was positively associated with the risk of CKD in a Chinese population with hypertension. This positive association was only significant in patients with BMI ≥24 kg/m^2^ who did not smoke, not in those with BMI <24 kg/m^2^ who smoked. These findings suggest that BMI and smoking status are significant effect modifiers.

Previous studies have indicated that dyslipidemia is related to renal insufficiency (14, 15); however, most are limited to traditional lipid indices, such as TG and HDL-C (16, 17). Few studies have investigated the relationship between unconventional lipid profiles and kidney disease. Qi et al. (18) designed a prospective cohort study of 3,909 participants with normal eGFR and a baseline age of ≥40 years, and the results showed that elevated blood lipid levels during follow-up increased the risk of eGFR decline. Higher RC levels are associated with the early progression of renal injuries. A cross-sectional study conducted by Marcelino et al. (19) involving 395 non-diabetic individuals who did not receive statins showed that the RC level of patients with CKD increased and was positively associated with the CKD stage. A cross-sectional study of 146 participants with type 2 diabetes at different CKD stages showed that lower renal function was associated with increased concentrations of CM, VLDL, or both (20). Apolipoprotein (apo) B48 is a specific marker of CM. A study of 101 participants with diabetic nephropathy of different stages showed that plasma apob48 levels increased with the progression of diabetic nephropathy (21). In contrast, a prospective cohort study by Rahman et al. showed no independent association between VLDL cholesterol levels and kidney disease progression (22). Some of these results were consistent with our conclusions, whereas others were not. The reason for this may be the different selections of race, population, and sample sizes. Yan et al. (23) conducted a cross-sectional study of 7,356 participants aged ≥40 years in China showing that a higher RC level is an independent risk factor for CKD (OR: 1.344, 95% CI: 1.097–1.648). This result is mostly consistent with the results of our study. However, as a high-risk group for CKD, patients with hypertension need to be considered as a special population to analyze the relationship between RC levels and CKD. Previous studies have confirmed that RC is a powerful lipid component that promotes atherosclerosis, and RC can act on the arterial wall through oxidative stress and low-grade inflammation, resulting in endothelial dysfunction and atherosclerosis, which can lead to the development of ASCVD and CKD (6, 24, 25). Although previous studies have discussed the relationship between RC levels and CKD, no study has investigated this association in populations with hypertension.

As a risk factor for CKD, a high TG level is related to the occurrence and progression of CKD. Hypertriglyceridemia is the prevalent feature of lipid metabolism disorders in patients with CKD. Therefore, we investigated the relationship between RC levels and CKD under normal TG levels and concluded that even if the TG level was normal, an increase in RC level was independently and positively associated with the risk of CKD. In further subgroup analysis, the positive association was more significant for people with BMI ≥24 kg/m^2^ (**Figure S2**).

In addition, we found that BMI and smoking status significantly modified the relationship between RC levels and CKD in a Chinese population with hypertension. With the increase in RC levels, participants with BMI ≥24 kg/m^2^ who did not smoke had a greater risk of CKD. For overweight or obese populations, the possible mechanisms are as follows. First, obesity can cause abnormal lipid metabolism in the human body, including an increase in TG concentration (26). This will increase the RC level and, thus, the risk of CKD. Second, being overweight or obese is a risk factor for developing CKD (27). Its impacts on renal function may be associated with comorbidities, such as diabetes or hypertension. However, it also leads to inflammation, oxidative stress, activation of the renal angiotensin-aldosterone system, and insulin resistance through the production of hormones, such as adiponectin, leptin, and resistin, resulting in increased glomerular hypertension and permeability, and ultimately, CKD (28–30). In previous studies, the effect of smoking on eGFR was unclear. Some studies have suggested that smoking is negatively correlated with eGFR, whereas others have suggested that smoking could increase eGFR (31–33). In our study, CKD was defined as eGFR less than 60 ml/min·1.73 m^2^; therefore, current non-smokers had a higher risk of CKD because non-smokers had lower eGFR values than smokers. This may be because of the following mechanisms. First, previous literature reviews have shown that current smokers tend to weigh less than non-smokers. It is well known that the source of creatinine in serum is muscle cells; therefore, in earlier studies, it was also found that the serum creatinine content in smokers was low (34, 35). However, the lower serum creatinine level in smokers may not simply be explained by the fact that smokers have less body fat and muscle mass, as the lower serum creatinine level remains even after adjusting for BMI (36). Consequently, smokers may have increased creatinine excretion, thereby increasing the eGFR (37). Second, smoking causes recurrent transient decreases in renal plasma flow and eGFR. This small recurrent transient renal hypoperfusion may damage some glomeruli and, thus, lead to aging of the peribulbar blood vessels and glomeruli and result in residual glomerular compensatory hypertrophy and hyperfiltration, ultimately leading to an elevated eGFR (38, 39). However, this compensatory increase in eGFR is limited, and as smoking volume and duration increase, renal function will eventually be severely impaired. In addition, previous studies have confirmed that smoking can damage kidney function. Perhaps, when smoking seriously damages kidney function (40, 41), the damage caused by increased RC on kidney function is not obvious. However, no definitive conclusions have been drawn regarding the effect of smoking on eGFR. According to existing research results, we believe that smokers have an increased compensatory eGFR in the early stages and a downward trend in eGFR in the latter stages owing to the aggravated impairment of renal function. Further research is required to explore this relationship in detail.

As a lipid index, RC has been studied more in cardiovascular diseases; however, studies on the relationship between RC levels and CKD have not received much attention in the past, let alone in individuals with hypertension. The guideline of the European Atherosclerosis Association (42) defines a high RC level as a fasting RC level ≥0.8 mmol/L and/or postprandial RC level ≥0.9 mmol/L; however, our research found that RC level ≥0.83 mmol/L significantly increased the risk of CKD, (OR, 1.40; 95% CI: 1.21–1.62), which suggests that reducing RC levels below 0.8 can reduce the risk of cardiovascular disease and CKS in both the general population and the population with hypertension. In our future clinical studies, we aim to calculate the value of RC levels using a simple formula and evaluate whether further lipid-lowering treatments are required. Whether a further reduction in RC levels can reduce the incidence rate requires further prospective research.

## 5 Study strengths and limitations

To the best of our knowledge, this is the first study to investigate the association between RC levels and CKD in a Chinese population with hypertension. However, this study has several limitations. First, we could not establish a causal relationship between RC levels and CKD because of the cross-sectional nature of the study. Second, the study population included participants with hypertension from rural areas of southern China who were aged over 18 years. Therefore, the results of this study cannot be generalized to individuals of other ages, regions, or disease types.

## 6 Conclusion

In the present study, we observed an independent positive association between elevated RC levels and CKD risk in a Chinese population with hypertension. This relationship seems to be more significant in patients with BMI ≥24 kg/m^2^ and current non-smokers. RC ≥0.83 mmol/L seems to be a rough cut-off point, which can be used to suggest that patients with hypertension need further lipid-lowering treatment. However, further studies are required to verify this. Our results are significant for clinical lipid management in patients with hypertension.

## Data availability statement

The raw data supporting the conclusions of this article will be made available by the authors, without undue reservation.

## Ethics statement

The studies involving human participants were reviewed and approved by the ethics committee of the Institute of biomedical research of Anhui Medical University. The patients/participants provided their written informed consent to participate in this study.

## Author contributions

TY, HB, and XC conceived and designed the study. TY, CD, and YX contributed to statistical analysis. TY drafted the manuscript. All authors contributed to the data collection and reviewed/edited the manuscript’s important intellectual content. All authors contributed to the article and approved the submitted version.

## Supplementary material

Supplementary Figure 1 Flow chart of participants.
