# Supplementary material for: Association between remnant cholesterol and chronic kidney disease in Chinese hypertensive patients
Source: Front Endocrinol (Lausanne). 2023 Jun 21;14:1189574. doi: 10.3389/fendo.2023.1189574 (PMC10321593; doi:10.3389/fendo.2023.1189574)
Supplement: Supplementary Figure 1 — Flow chart of participants. [file DataSheet_1.docx]

**Table S1. Association between RC and eGFR in different models**

| RC, mmol/L | Participants, n | eGFR, ml/min/1.73 m² | Crude model | |  | Model I | | |  | Model II | |
| --- | --- | --- | --- | --- | --- | --- | --- | --- | --- | --- | --- |
|  |  |  | β (95% CI) | *P* value |  | β (95% CI) | *P* value | |  | β (95% CI) | *P* value |
| RC Z score | 13024 | 88.3 ± 20.1 | -1.74 (-2.09, -1.40) | <0.001 |  | -2.09 (-2.39, -1.79) | <0.001 | |  | -1.97 (-2.29, -1.65) | <0.001 |
| Quartiles |  |  |  |  |  |  |  |  | |  |  |
| Q1(<0.41) | 3150 | 91.4 ± 20.6 | Ref. |  |  | Ref. |  |  | | Ref. |  |
| Q2(0.41-0.63) | 3319 | 88.5 ± 19.8 | -2.87 (-3.84, -1.90) | <0.001 |  | -3.00 (-3.84, -2.16) | <0.001 | |  | -2.95 (-3.79, -2.11) | <0.001 |
| Q3(0.63-0.83) | 3205 | 87.4 ± 19.3 | -3.99 (-4.97, -3.01) | <0.001 |  | -4.09 (-4.94, -3.24) | <0.001 | |  | -4.05 (-4.90, -3.20) | <0.001 |
| Q4(≥0.83) | 3350 | 86.0 ± 20.2 | -5.38 (-6.35, -4.40) | <0.001 |  | -6.45 (-7.29, -5.60) | <0.001 | |  | -6.35 (-7.20, -5.51) | <0.001 |
| *P* for trend |  |  |  | <0.001 |  |  | <0.001 | |  |  | <0.001 |
| Categories |  |  |  |  |  |  |  | |  |  |  |
| Q1-Q3(<0.83) | 9674 | 89.07 ± 19.96 | Ref. |  |  | Ref. |  | |  | Ref. |  |
| Q4(≥0.83) | 3350 | 86.00 ± 20.22 | -3.07 (-3.86, -2.28) | <0.001 |  | -4.04 (-4.73, -3.36) | <0.001 | |  | -3.97 (-4.66, -3.29) | <0.001 |

Crude model was adjusted for None; Model I was adjusted for age, sex, BMI, WC; Model II was adjusted for age, sex, BMI, WC, SBP, DBP, TG, smoking status, alcohol drinking status, Hcy, diabetes, stroke, coronary heart disease, antihypertensive drugs

Abbreviations: RC, Remnant cholesterol; OR, Odd ratio; 95% CI, 95% Confidence interval; TG, triglycerides; Hcy, homocysteine

**Table S2. Association between RC and CKD in different models in individuals with normal TG level**

| RC, mmol/L | Participants, n | Events, n (%) | Crude model | |  | Model I | |  | Model II | |
| --- | --- | --- | --- | --- | --- | --- | --- | --- | --- | --- |
|  |  |  | OR (95% CI) | *P* value |  | OR (95% CI) | *P* value |  | OR (95% CI) | *P* value |
| RC Z score | 7749 | 761 (9.82) | 1.13 (1.06, 1.21) | <0.001 |  | 1.08 (1.01, 1.17) | 0.034 |  | 1.11 (1.02, 1.19) | 0.011 |
| Categories |  |  |  |  |  |  |  |  |  |  |
| Q1(<0.33) | 1926 | 157 (8.15) | Ref. |  |  | Ref. |  |  | Ref. |  |
| Q2(0.33-0.53) | 1845 | 182 (9.86) | 1.23 (0.99, 1.54) | 0.066 |  | 1.14 (0.90, 1.44) | 0.273 |  | 1.17 (0.92, 1.49) | 0.205 |
| Q3(0.53-0.71) | 2040 | 191 (9.36) | 1.16 (0.93, 1.45) | 0.178 |  | 1.09 (0.86, 1.37) | 0.473 |  | 1.11 (0.87, 1.41) | 0.393 |
| Q4(≥0.71) | 1938 | 231 (11.92) | 1.52 (1.23, 1.89) | <0.001 |  | 1.32 (1.05, 1.65) | 0.017 |  | 1.42 (1.12, 1.79) | 0.004 |
| *P* for trend |  |  |  | <0.001 |  |  | 0.029 |  |  | 0.007 |

Crude model was adjusted for None; Model I was adjusted for age, sex, BMI, WC; Model II was adjusted for age, sex, BMI, WC, SBP, DBP, smoking status, alcohol drinking status, Hcy, diabetes, stroke, coronary heart disease, antihypertensive drugs

Abbreviations: RC, Remnant cholesterol; OR, Odd ratio; 95% CI, 95% Confidence interval; Hcy, homocysteine

**Table S3. Association between RC and CKD** **after further adjustment for lipid indices**

| RC, mmol/L | Participants, n | Events, n (%) | Model I | |  |  | Model II | |
| --- | --- | --- | --- | --- | --- | --- | --- | --- |
|  |  |  | OR (95% CI) | *P* value |  |  | OR (95% CI) | *P* value |
| RC Z score | 13024 | 1243 (9.5) | 1.15 (1.08, 1.23) | <0.001 |  |  | 1.17 (1.09, 1.25) | <0.001 |
| Quartiles |  |  |  |  |  |  |  |  |
| Q1(<0.41) | 3150 | 257 (8.2) | Ref. |  |  |  | Ref. |  |
| Q2(0.41-0.63) | 3319 | 299 (9.0) | 1.12 (0.93, 1.35) | 0.247 |  |  | 1.10 (0.91, 1.33) | 0.325 |
| Q3(0.63-0.83) | 3205 | 297 (9.3) | 1.15 (0.95, 1.39) | 0.118 |  |  | 1.14 (0.94, 1.38) | 0.178 |
| Q4(≥0.83) | 3350 | 390 (11.6) | 1.53 (1.26, 1.86) | <0.001 |  |  | 1.57 (1.29, 1.91) | <0.001 |
| *P* for trend |  |  |  | <0.001 |  |  |  | <0.001 |
| Categories |  |  |  |  |  |  |  |  |
| Q1-Q3(<0.83) | 9674 | 853 (8.8) | Ref. |  |  |  | Ref. |  |
| Q4(≥0.83) | 3350 | 390 (11.6) | 1.40 (1.20, 1.62) | <0.001 |  |  | 1.44 (1.24, 1.68) | <0.001 |

Model I was adjusted for age, sex, BMI, WC, SBP, DBP, TG, smoking status, alcohol drinking status, Hcy, diabetes, stroke, coronary heart disease, antihypertensive drugs; Model II was adjusted for age, sex, BMI, WC, SBP, DBP, TG, smoking status, alcohol drinking status, Hcy, diabetes, stroke, coronary heart disease, antihypertensive drugs, TC residue, HDL-C residue, LDL-C residue;

Abbreviations: OR, odd ratio; 95% CI, 95% confidence interval; RC, remnant cholesterol; BMI, body mass index; WC, waist circumference; SBP, systolic blood pressure; DBP, diastolic blood pressure; TG, triglyceride; Hcy, homocysteine; HDL-C, high-density lipoprotein cholesterol; LDL-C, low-density lipoprotein cholesterol


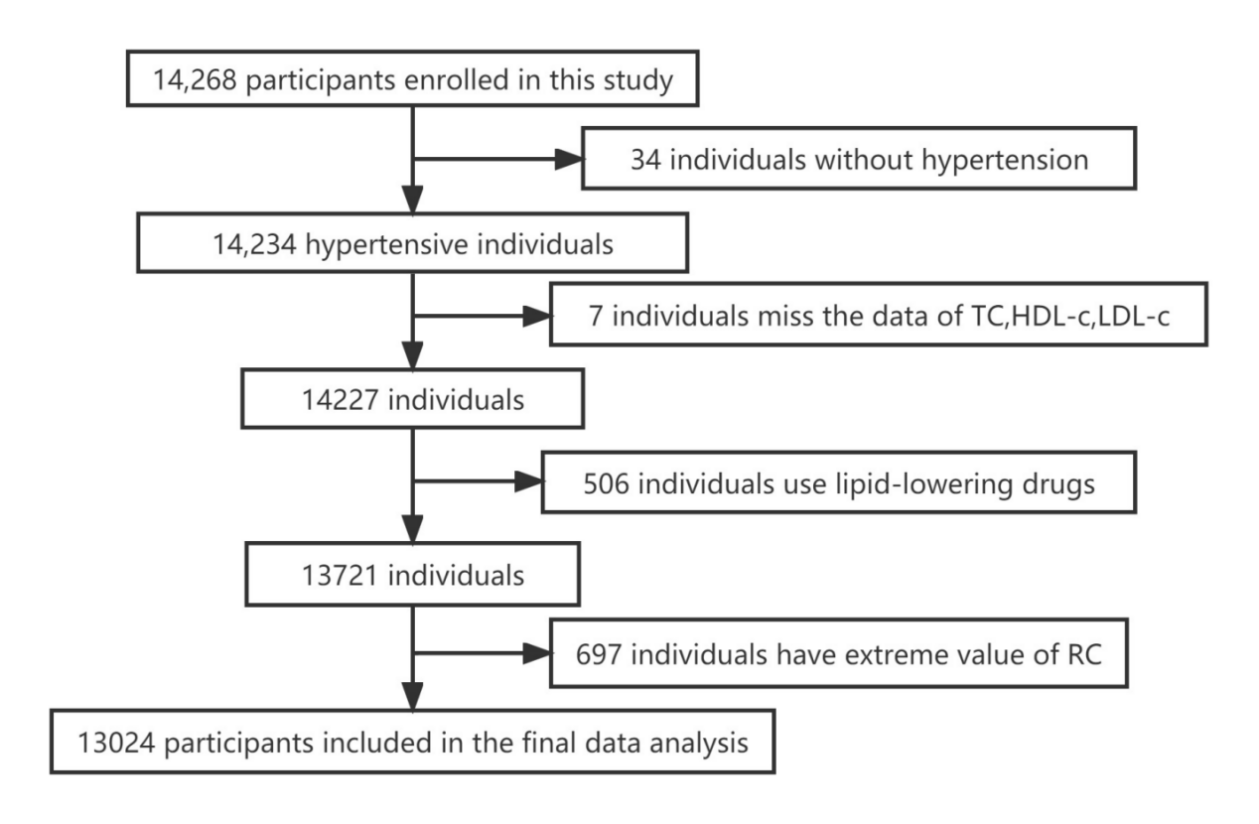


**Figure S1. Flow chart of participants**
